# Supplementary material for: Substitution of arginine 219 by glycine compromises stability, dimerization, and catalytic activity in a G6PD mutant
Source: Commun Biol. 2023 Dec 9;6:1245. doi: 10.1038/s42003-023-05599-z (PMC10709299; doi:10.1038/s42003-023-05599-z)

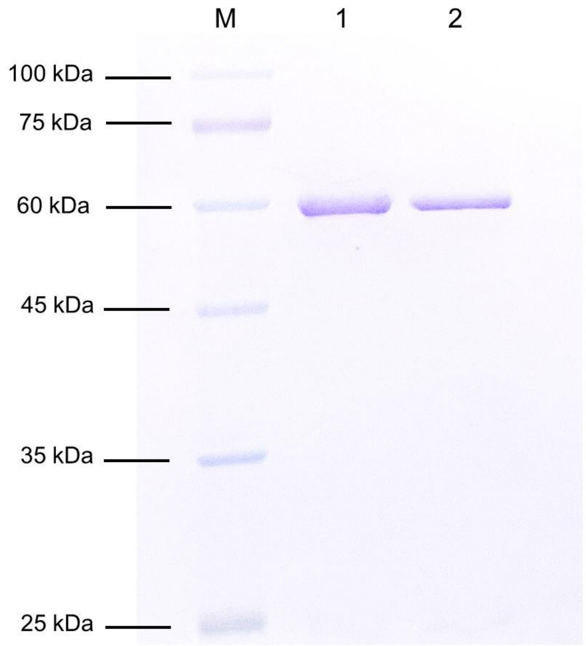

Supplementary Figure 1. SDS-PAGE analysis of recombinant G6PD proteins. Lane M, molecular mass marker proteins; lane 1, G6PD WT and lane 2, G6PD Meyer.

Uncropped file for supplementary figure 1

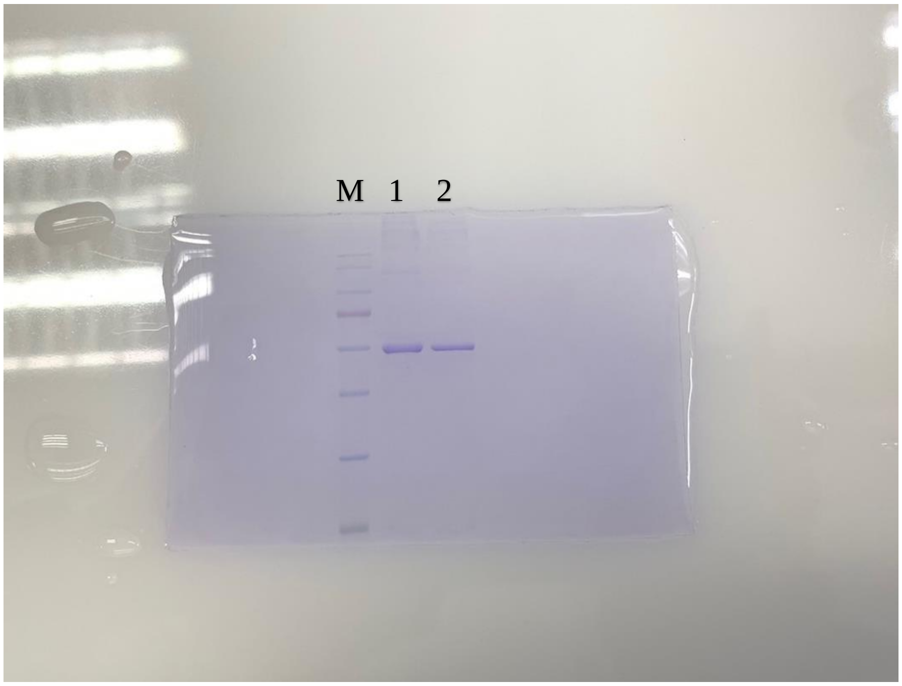

Supplement: Supplementary file 1 — Supplementary Information [file 42003_2023_5599_MOESM1_ESM.pdf]
